# Metabolic Reprogramming and Immunometabolic Dysregulation in Diabetic Kidney Disease: From Pathogenesis to Precision Multi-target Therapies

**DOI:** 10.34133/research.1386

**Published:** 2026-08-04

**Authors:** Ziyue Zhang, Yilun Qu, Xiaochen Wang, Yuwei Ji, Jin Yao, Weizhu Deng, Guannan Sun, Hua Xu, Xiangmei Chen, Quan Hong

**Affiliations:** ^1^ Department of Nephrology, Chinese PLA General Hospital; Chinese PLA Institute of Nephrology; State Key Laboratory of Kidney Diseases; National Clinical Research Center of Kidney Diseases, Beijing 100853, China.; ^2^ Academy of Military Medical Sciences, Beijing 100850, China.

## Abstract

Diabetic kidney disease, the leading cause of end-stage kidney disease worldwide, involves complex interactions beyond classical hemodynamic and oxidative stress pathways. Recent advances emphasize metabolic reprogramming in renal cells—characterized by mitochondrial dysfunction, impaired fatty acid oxidation, lipotoxicity, and glycolytic shifts—as upstream drivers of cellular injury and fibrosis. Single-cell RNA sequencing reveals profound immunometabolic heterogeneity, including dynamic macrophage subpopulations (e.g., proinflammatory early states transitioning to TREM2^hi^/MRC1^hi^ lipid-associated phenotypes) and T helper 17/regulatory T imbalance, which amplify inflammation via bidirectional crosstalk with podocytes, tubular cells, and mesangial cells. Interorgan axes, particularly gut dysbiosis and uremic toxin accumulation, further perpetuate immune dysregulation. This review integrates these insights to propose precision strategies targeting mitochondrial homeostasis, ferroptosis inhibition, glycolytic blockade in immune cells, and multimodal therapies (e.g., combination strategies integrating sodium–glucose cotransporter 2 inhibitors with immunometabolic modulators). Multi-omics integration and spatial transcriptomics hold promise for individualized and mechanism-guided interventions to halt diabetic kidney disease progression.

## Introduction

The global burden of diabetes mellitus continues to escalate, establishing it as a leading cause of chronic kidney disease (CKD) and end-stage renal disease. Over the past 3 decades, the incidence of CKD associated with type 2 diabetes has surged by 74%, while the proportion of end-stage renal disease cases attributable to type 2 diabetes rose from 22.1% in 2000 to 31.3% by 2015 [1]. According to the 2017 Global Burden of Disease study, mortality rates associated with diabetic kidney disease (DKD)-related CKD have increased substantially, with approximately 1.2 million deaths annually linked to its complications [2]. Despite current management strategies focusing on glycemic control, blood pressure regulation, and renoprotective interventions, existing pharmacotherapies remain insufficient to halt renal fibrosis and progressive loss of kidney function. Consequently, there is an urgent clinical need to identify novel therapeutic strategies targeting the core pathological drivers of the disease [3].

Traditionally, DKD pathogenesis has been framed around hemodynamic abnormalities, activation of the renin–angiotensin–aldosterone system (RAAS), glucotoxicity, oxidative stress, and inflammation. However, this classical paradigm is increasingly recognized as incomplete. It fails to explain the pronounced interindividual heterogeneity in disease progression, particularly the observation that some patients experience rapid renal decline despite stringent metabolic and blood pressure control. Moreover, conventional models often treat inflammation and fibrosis as parallel downstream outcomes rather than mechanistically interconnected processes and inadequately characterize the context-dependent roles of immune cells in disease progression. These conceptual limitations have hindered the identification of effective molecular targets.

To bridge these critical gaps, recent investigations have expanded the mechanistic framework of DKD beyond conventional paradigms. It is now recognized that the disease involves a complex regulatory network characterized by macrophage heterogeneity, metabolic reprogramming, and intricate crosstalk between immune cells and renal-resident cells [4–6]. These emerging mechanisms suggest that metabolic state and immune function are tightly interwoven determinants of renal injury. A systematic dissection of these interconnected pathways is therefore essential to identify key molecular nodes that transcend traditional single-pathway models. Such insights may enable precision-guided and multi-target therapeutic strategies. This review aims to integrate these emerging concepts to provide a theoretical framework for overcoming current therapeutic bottlenecks, fostering the development of precision medicine approaches, and improving clinical outcomes for patients with DKD [7]. This review aims to integrate these emerging insights to provide a theoretical framework for bypassing current therapeutic bottlenecks, fostering the development of precision medicine strategies, and ultimately improving long-term clinical outcomes for patients with DKD.

## Renal Pathological Manifestations

Pathological alterations in DKD manifest as structural remodeling involving the entire nephron. In the classic albuminuric trajectory, early lesions are predominantly glomerular, characterized by glomerular hypertrophy, diffuse glomerular basement membrane thickening, mesangial matrix expansion, and, in advanced disease, the formation of Kimmelstiel–Wilson nodules [8]. Within this trajectory, podocyte injury is recognized as a major contributor to early filtration barrier dysfunction and promotes progressive mesangial matrix accumulation and glomerulosclerosis. However, current evidence suggests that podocyte injury should not be interpreted as the universal initiating event across all DKD phenotypes. Rather, DKD comprises heterogeneous pathological trajectories in which glomerular and tubulointerstitial injuries may emerge with different temporal predominance and relative contributions to disease progression [9–11].

Tubulointerstitial injury represents another major pathological axis that may dominate in specific DKD phenotypes, particularly nonalbuminuric DKD. It is characterized by tubular epithelial cell (TEC) hypertrophy and atrophy, tubular basement membrane thickening, inflammatory cell infiltration, and interstitial fibrosis. Importantly, the extent of interstitial fibrosis correlates more strongly with glomerular filtration rate decline and renal prognosis than glomerulosclerosis does [12]. Moreover, the recognition of nonalbuminuric DKD and the emergence of early tubular injury biomarkers such as kidney injury molecule-1 indicate that tubulointerstitial damage can occur before overt albuminuria and may dominate the clinical course in a subset of patients. Collectively, current evidence supports the view that glomerular/podocyte injury and tubulointerstitial injury represent complementary and partially independent pathological pathways that converge to drive nephron loss and progressive kidney dysfunction in DKD [13,14].

Clinically, these distinct pathological trajectories are reflected by heterogeneous patterns of disease progression. While increasing albuminuria remains a hallmark of the classical albuminuric pathway and reflects progressive impairment of the glomerular filtration barrier, the emergence of nonalbuminuric DKD demonstrates that marked tubulointerstitial injury can precede or occur independently of overt albuminuria. Nevertheless, both trajectories converge on a common end point characterized by progressive nephron loss, manifested clinically as an irreversible decline in estimated glomerular filtration rate (eGFR). This functional deterioration results from the combined effects of glomerulosclerosis, tubulointerstitial fibrosis, vascular remodeling, and nephron dropout [15]. Consequently, contemporary pathological assessment has evolved from a predominantly glomerulo-centric framework toward a holistic evaluation of nephron-wide structural remodeling and overall renal integrity [16] (Table 1).

**Table 1.** Key pathological features of DKD and their related mechanisms

Pathological feature	Primary cell types involved	Key underlying mechanisms	Associated clinical/biomarker sign
Thickening of the GBM	Podocytes, endothelial cells	High blood sugar, activation of RAAS, AGEs, TGF-β signaling	Proteinuria, decreased GFR
Mesangial matrix expansion/nodular sclerosis	Mesangial cells, podocytes	RAAS activation, TGF-β signaling, inflammation, oxidative stress, GBM renewal disorder	Proteinuria, GFR decline
Podocyte foot process fusion/fusion failure	Podocytes	Cardiovascular stress, activation of RAAS, oxidative stress, metabolic reprogramming	Proteinuria (especially albuminuria)
Atrophy/apoptosis of renal tubular epithelial cells	Proximal renal tubular cells	Ischemia, mitochondrial dysfunction, FAO disorder, lipotoxicity	Decreased GFR, elevated urinary KIM-1/NGAL
Renal interstitial fibrosis	Fibroblasts, renal tubular cells, macrophages	TGF-β signaling, inflammation (NLRP3 activation), hypoxia, EMT	Progressive decline in GFR, ESRD

DKD, diabetic kidney disease; GBM, glomerular basement membrane; ESRD, end-stage renal disease; RAAS, renin–angiotensin–aldosterone system; KIM-1, kidney injury molecule-1; TGF-β1, transforming growth factor-β1; AGE, advanced glycation end product; FAO, fatty acid oxidation; GFR, glomerular filtration rate; EMT, epithelial–mesenchymal transition; NLRP3, NOD-like receptor family pyrin domain containing 3; NGAL, neutrophil gelatinase-associated lipocalin

## Advances in Classical Pathogenic Mechanisms of DKD

DKD progression is driven by classical pathways including hemodynamic abnormalities, RAAS activation, glucotoxicity, oxidative stress, and inflammation. Recent research has unveiled more refined molecular regulatory networks underlying these processes [17].

### Hemodynamic abnormalities: The molecular basis of glomerular hyperfiltration

Glomerular hyperfiltration represents a pivotal early feature of DKD, primarily driven by an imbalance in afferent and efferent arteriolar resistance that increases intraglomerular capillary pressure. Afferent arteriolar dilation results from impaired tubuloglomerular feedback: Under hyperglycemic conditions, increased sodium–glucose cotransporter 2 (SGLT2) activity enhances proximal tubular glucose and sodium reabsorption, reducing macula densa sodium sensing and promoting afferent vasodilation [18,19]. Conversely, efferent arteriolar constriction is primarily mediated by angiotensin II (Ang II). This combination of afferent dilation and efferent constriction elevates intraglomerular hydrostatic pressure, generating mechanical stress (“barotrauma”) that contributes to podocyte and endothelial injury, thereby promoting proteinuria and glomerulosclerosis [20,21].

### Dual roles of the RAAS

The RAAS exhibits a dual-axis regulatory imbalance in DKD [22]. The classical angiotensin-converting enzyme (ACE)/Ang II/angiotensin Ⅱ receptor type 1 axis is excessively activated under hyperglycemic conditions. This activation recruits downstream phospholipase C/Ca^2+^ signaling, as well as mitogen-activated protein kinase and nuclear factor κB (NF-κB) pathways, promoting the expression of proinflammatory and profibrotic mediators such as transforming growth factor-β1 (TGF-β) and monocyte chemoattractant protein-1 (MCP-1) , thereby contributing to mesangial matrix expansion and podocyte injury. Conversely, the ACE2/Ang-(1–7)/Mas protective axis counterbalances these pathogenic effects. ACE2 cleaves Ang II into Ang-(1–7), which subsequently binds to the Mas receptor and activates the phosphatidylinositol 3-kinase/Akt/endothelial nitric oxide synthase pathway, enhancing NO production and promoting vasodilation [23,24]. Concurrently, this protective axis suppresses nicotinamide adenine dinucleotide phosphate (NADPH) oxidase (NOX) activity and reactive oxygen species (ROS) generation while inhibiting NF-κB nuclear translocation, thereby exerting anti-inflammatory and antifibrotic effects. During DKD progression, renal ACE2 expression is reduced and ACE2 shedding is increased, resulting in diminished activity of the protective axis and impaired counter-regulation of the classical pathogenic axis [25]. Consequently, emerging therapeutic approaches are exploring ACE2 activation or recombinant ACE2 supplementation as potential strategies to restore homeostatic balance between these dual axes, extending beyond conventional RAAS blockade with ACE inhibitors and angiotensin receptor blockers [26].

### Hyperglycemia-induced cellular damage pathways

Persistent hyperglycemia diverts excess glucose into 4 major interconnected collateral pathways that collectively contribute to glucotoxicity [27]:

1. The advanced glycation end products (AGEs) pathway: Accumulation of nonenzymatic AGEs within extracellular matrices (ECMs), including the basement membrane, promotes matrix stiffening. Concurrently, AGE engagement with its receptor activates NOX to generate ROS, which subsequently activates NF-κB signaling and up-regulates inflammatory molecules such as intercellular adhension molecule 1, thereby contributing to chronic inflammatory injury [28].
2. The protein kinase C (PKC) pathway: Excess glycolytic intermediates promote the synthesis of 1,2-diacylglycerol, which chronically activates PKC-β and PKC-α isoforms. Downstream phosphorylation events induce vascular endothelial growth factor (VEGF) up-regulation, contributing to increased vascular permeability and proteinuria, promote TGF-β1 synthesis and ECM deposition, and impair endothelial nitric oxide synthase function, leading to endothelial dysfunction [29].
3. The polyol pathway: Aldose reductase reduces glucose to sorbitol, utilizing NADPH as a cofactor. This pathway induces cellular injury through 2 major mechanisms: First, excessive NADPH consumption impairs glutathione reductase activity, reducing glutathione regeneration and weakening cellular antioxidant capacity; second, intracellular sorbitol accumulation increases osmotic stress, thereby contributing to cellular edema and injury [30].
4. The hexosamine pathway: Fructose-6-phosphate is diverted into the hexosamine biosynthetic pathway, where it is catalyzed by the rate-limiting enzyme glutamine:fructose-6-phosphate amidotransferase to generate uridine diphosphate-N-acetylglucosamine (GlcNAc). Driven by O-GlcNAc transferase, transcription factors such as Sp1 and NF-κB undergo aberrant O-GlcNAc modification, promoting transcriptional activation of profibrotic genes, including plasminogen activator inhibitor-1 and TGF-β1 [31].

### Oxidative stress and inflammation: The core malignant cycle

**Fig. 1.**
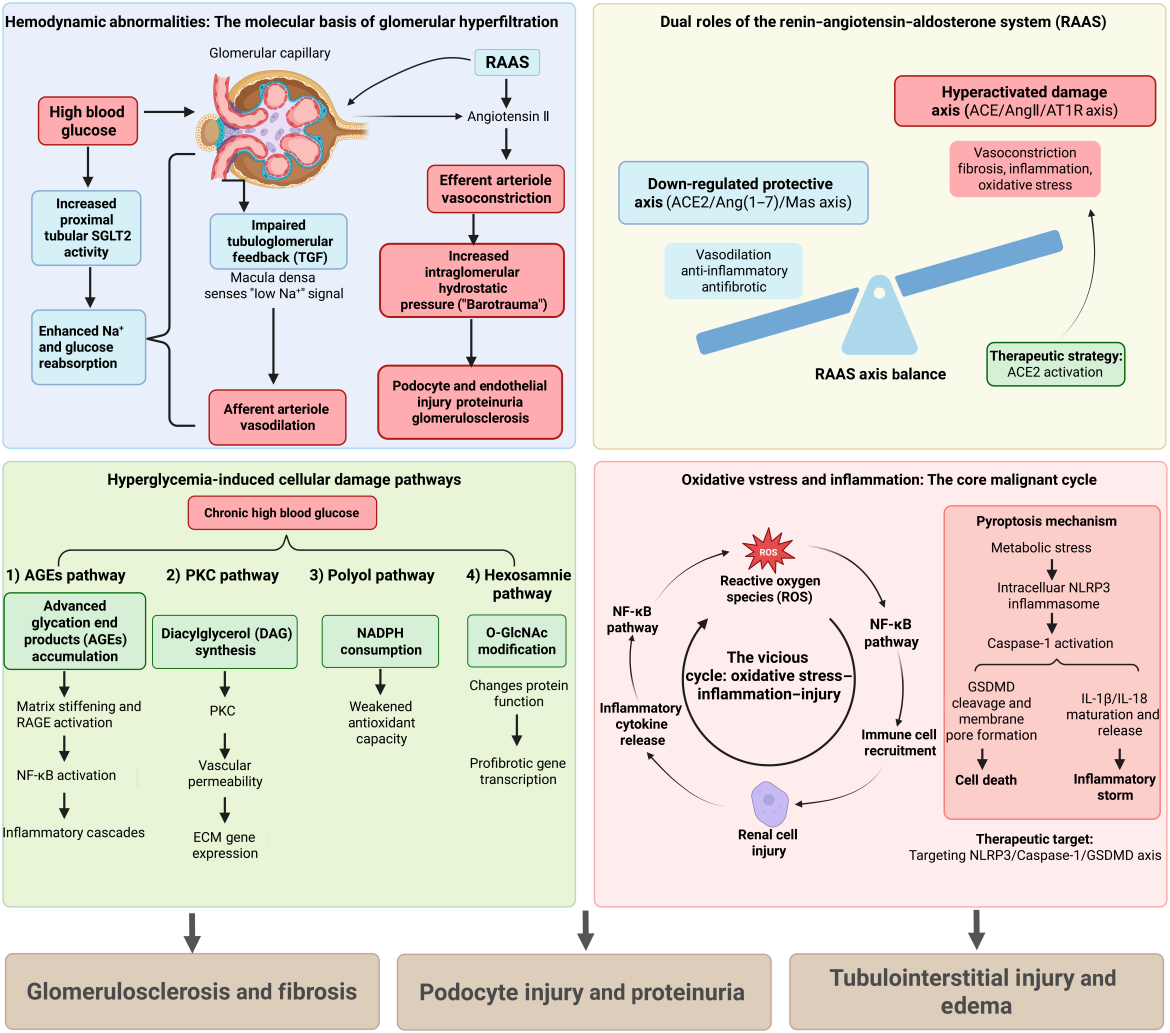
Multifaceted pathogenesis of diabetic kidney disease (DKD). The progression of DKD is driven by a synergistic interplay of hemodynamic abnormalities, in which SGLT2-mediated sodium reabsorption and tubuloglomerular feedback (TGF) impairment lead to afferent vasodilation and renin–angiotensin–aldosterone system (RAAS)-induced efferent vasoconstriction, causing glomerular hyperfiltration and barotrauma. This is exacerbated by RAAS imbalance, favoring the proinflammatory angiotensin-converting enzyme (ACE)–Ang II–angiotensin Ⅱ receptor type 1 (AT1R) axis over the protective ACE2–Mas axis. Concurrently, hyperglycemia triggers 4 metabolic pathways (advanced glycation end products [AGEs], protein kinase C [PKC], polyol, and hexosamine) that induce cellular damage and matrix remodeling. These processes culminate in a vicious cycle of oxidative stress and inflammation, ultimately triggering NOD-like receptor family pyrin domain containing 3 (NLRP3)-mediated pyroptosis and renal cell injury, highlighting ACE2 and gasdermin D (GSDMD) as potential therapeutic targets. Figure created with BioRender.com.

Oxidative stress and inflammation form a self-reinforcing pathogenic loop in DKD. Excessive ROS production promotes redox-sensitive activation of pathways such as NF-κB, thereby inducing inflammatory cytokine production and facilitating immune cell recruitment [32]. Recent advances have identified pyroptosis as an important mechanism linking metabolic stress to inflammatory amplification. Metabolic stressors, including oxidative stress and mitochondrial dysfunction, activate the intracellular NOD-like receptor family pyrin domain containing 3 (NLRP3) inflammasome, leading to Caspase-1 activation. Activated Caspase-1 cleaves gasdermin D to generate membrane pores that drive pyroptotic cell death, while simultaneously promoting the maturation and release of interleukin-1β (IL-1β) and IL-18. Targeting the NLRP3/Caspase-1/gasdermin D pyroptosis axis represents a potential therapeutic strategy to attenuate inflammatory amplification and renal injury in DKD [33,34] (Fig. 1).

## Metabolic Reprogramming

Beyond classical pathways, a new paradigm centered on cellular metabolic reprogramming has emerged. This concept posits that the fundamental remodeling of metabolic programs in renal cells under diabetic conditions represents a core upstream event driving cellular dysfunction and tissue fibrosis [35,36].

### Energy metabolic reprogramming: From fatty acid oxidation to glycolysis

A healthy kidney is an energy-consuming organ, with its energy demand being second only to that of the heart. Particularly, the TECs of the proximal renal tubules, which undertake the majority of substance reabsorption tasks, have mitochondria-rich cytoplasm and almost entirely rely on efficient mitochondrial fatty acid oxidation (FAO) to generate the large amount of adenosine triphosphate (ATP) required to maintain ion pump function [36].

**Fig. 2.**
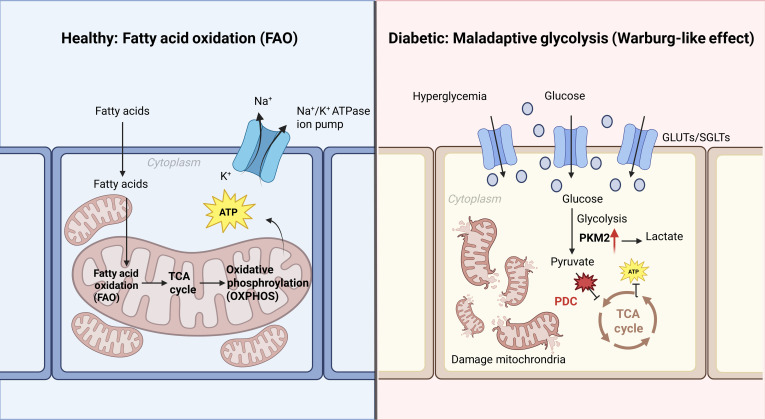
Metabolic reprogramming in renal tubular epithelial cells under high-glucose conditions. In the healthy state (left), renal tubules primarily utilize mitochondrial fatty acid oxidation (FAO) and oxidative phosphorylation (OXPHOS) to meet the high energy demands of reabsorption. Conversely, under high-glucose conditions (right), an important metabolic shift occurs: FAO is suppressed, and mitochondrial dysfunction ensues. Increased glucose uptake via up-regulated sodium–glucose cotransporters 2 (SGLTs) and GLUTs, coupled with pyruvate dehydrogenase complex (PDC) inhibition, shunts pyruvate toward enhanced glycolysis and lactate production. This transition from efficient oxidative metabolism to glycolysis results in adenosine triphosphate (ATP) deficiency, contributing to tubular injury and the progression of diabetic kidney disease. Figure created with BioRender.com. TCA, tricarboxylic acid.

However, under diabetic pathological conditions, the energy metabolic program of TECs undergoes a dramatic and maladaptive shift. FAO is markedly suppressed, and cells instead become dependent on the substantially less efficient process of glycolysis for ATP production [35,36]. This metabolic transition from oxidative phosphorylation (OXPHOS) to glycolysis closely resembles the well-known “Warburg effect” observed in cancer cells [36]. Multiple mechanisms drive this shift. On one hand, hyperglycemia increases intracellular glucose availability through the up-regulation of glucose transporters (GLUTs and SGLTs), thereby promoting glycolytic flux. Pyruvate kinase M2 (PKM2), a key glycolytic enzyme, is up-regulated in glomerular podocytes, TECs, and perivascular cells under high-glucose conditions, facilitating the conversion of glucose to lactate and reducing reliance on mitochondrial OXPHOS [37–39]. On the other hand, the pyruvate dehydrogenase complex, a critical enzymatic link between glycolysis and the tricarboxylic acid (TCA) cycle, is inhibited under high-glucose conditions. This inhibition prevents pyruvate from entering mitochondria for efficient OXPHOS, thereby forcing cells to adopt glycolysis as a compensatory, yet energetically inferior, ATP-generating pathway [35,37] (Fig. 2).

### Mitochondrial dysfunction: The nexus of energy crisis and oxidative damage

**Fig. 3.**
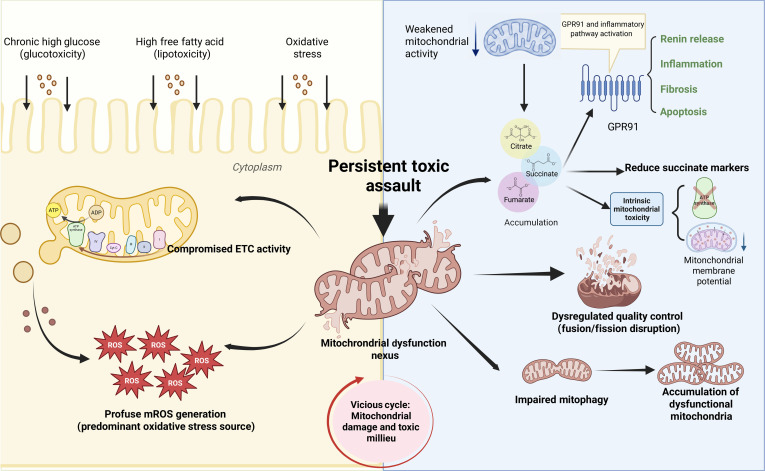
The pathophysiological cascade of the mitochondrial dysfunction nexus. Persistent toxic insults trigger a vicious cycle of impaired electron transport chain (ETC) activity and excessive mitochondrial reactive oxygen species (mROS) generation. This leads to the accumulation of metabolites (e.g., succinate), which activate inflammatory pathways via G protein-coupled receptor 91 (GPR91), while promoting mitochondrial quality control dysregulation and impaired mitophagy, ultimately resulting in cellular bioenergetic failure and apoptosis. Figure created with BioRender.com. ADP, adenosine diphosphate.

Mitochondria serve as the cellular “power plants” and occupy a central position in the metabolic reprogramming of DKD. Under persistent assault from glucotoxicity, lipotoxicity, and oxidative stress, mitochondrial function undergoes severe impairment. This dysfunction manifests primarily through the following dimensions:

First, the activity of the electron transport chain is compromised, leading to a substantial decline in ATP synthesis efficiency and precipitating a cellular “energy crisis”. Second, augmented electron leakage within the electron transport chain results in excessive generation of mitochondrial ROS, which constitutes a predominant source of oxidative stress in DKD [32]. Furthermore, the mitochondrial quality control system becomes dysregulated. This involves disruption of the dynamic equilibrium between mitochondrial fusion and fission, coupled with impairment of mitophagy—the process responsible for clearing damaged mitochondria. Consequently, dysfunctional mitochondria accumulate intracellularly, further exacerbating energy deficits and oxidative stress. Finally, attenuated mitochondrial activity leads to the accumulation of upstream metabolites, including citrate, succinate, and fumarate. These metabolites aggravate renal injury by activating the G protein-coupled receptor 91 receptor and various inflammatory pathways, promoting renin release, inflammation, fibrosis, and apoptosis [40]. Clinically, urinary succinate has been identified as an independent biomarker for DKD and is significantly correlated with albuminuria [41]. Notably, these metabolites exert intrinsic toxicity on mitochondria by inhibiting ATP synthase or reducing mitochondrial membrane potential. This establishes a vicious cycle: Mitochondrial dysfunction triggers oxidative stress and toxic metabolite accumulation, which in turn exacerbate mitochondrial damage, thereby amplifying and perpetuating cellular injury [42] (Fig. 3).

### Consequences of lipotoxicity and metabolite accumulation

The shift in cellular metabolic programs from FAO to glycolysis entails consequences far beyond diminished energy production efficiency. This transition orchestrates an intracellular “perfect storm” characterized by lipid overload and aberrant metabolite accumulation, which represents a pivotal mechanism translating upstream hyperglycemic signals into downstream cellular stress and fibrogenesis [35,36].

**Fig. 4.**
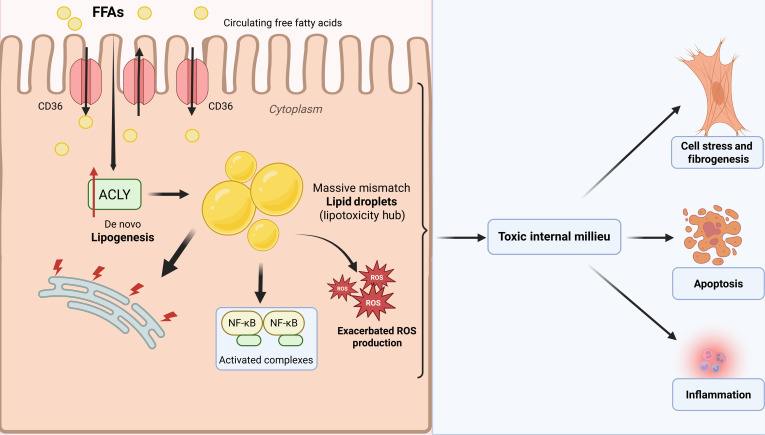
Mechanism of lipotoxicity-induced cellular damage and the toxic internal milieu. CD36-mediated lipid uptake and adenosine triphosphate-citrate lyase (ACLY)-driven de novo lipogenesis promote lipid droplet accumulation, establishing a central “lipotoxicity hub”. This accumulation triggers endoplasmic reticulum stress, nuclear factor κB (NF-κB) activation, and excessive reactive oxygen species (ROS) production. Collectively, these pathological processes establish a toxic internal milieu that promotes cellular stress, fibrogenesis, apoptosis, and chronic inflammation. Figure created with BioRender.com. FFA, free fatty acids.

The logic chain of this process is delineated as follows: First, although TECs cease utilizing fatty acids as a primary fuel for oxidative metabolism; their uptake of circulating fatty acids persists and may even increase due to the up-regulation of fatty acid transporters, such as CD36. Furthermore, ATP-citrate lyase, a rate-limiting enzyme in de novo lipogenesis, is up-regulated in DKD, promoting fatty acid synthesis and lipid accretion. This severe mismatch between uptake and consumption leads to the abnormal accumulation of unutilized fatty acids—including free fatty acids and triglycerides—within the cytoplasm of TECs in the form of lipid droplets [43]. This intracellular lipid overload exerts direct cytotoxic effects, a phenomenon termed lipotoxicity. Lipotoxicity induces cellular damage through multiple pathways, including the induction of endoplasmic reticulum (ER) stress, activation of proinflammatory signaling pathways, and enhanced production of ROS, ultimately culminating in apoptosis and fibrosis [44]. Concurrently, enhanced glycolysis coupled with a disrupted TCA cycle leads to abnormal intracellular accumulation of glycolytic and TCA intermediates, such as lactate and fumarate. These metabolites possess intrinsic biological activity, functioning as signaling molecules that directly activate proinflammatory and profibrotic pathways. Consequently, metabolic reprogramming places the cell in a highly toxic internal milieu by simultaneously triggering lipotoxicity and deleterious metabolite accumulation. This understanding positions the cellular metabolic state as a central hub that integrates hyperglycemic signals and transduces them into specific cellular stress responses—such as ER stress, inflammation, and apoptosis—that drive fibrosis and organ failure [35,36,44] (Fig. 4).

## Immunometabolism: An Emerging Frontier Linking Metabolism and Inflammation

Immunometabolism is an emerging interdisciplinary field investigating the reciprocal regulation between cellular metabolic pathways and immune function. A core tenet of this field is that the metabolic state of a cell shapes its functional phenotype. For instance, proinflammatory M1-type macrophages primarily rely on glycolysis to support rapid biosynthesis and effector functions, whereas anti-inflammatory M2-type macrophages depend on OXPHOS [43,45].

In the context of DKD, the local pathological microenvironment—characterized by hyperglycemia, hyperlipidemia, and hypoxia—remodels the metabolism of both resident renal cells and infiltrating immune cells. This environment drives macrophages and T cells toward a glycolysis-dependent, proinflammatory state, thereby markedly amplifying immune-mediated renal injury [46].

### Macrophage heterogeneity in DKD: From cellular taxonomy to immunometabolic state transitions

#### Single-cell atlases redefine macrophage heterogeneity beyond the M1/M2 paradigm

The advent of single-cell technologies has substantially reshaped the understanding of macrophage biology in DKD. Rather than supporting the traditional binary classification of classically activated (M1) and alternatively activated (M2) macrophages, single-cell RNA sequencing, single-nucleus RNA sequencing, and spatial transcriptomics (ST) collectively reveal that renal macrophages exist along a continuum of transcriptional and functional states that dynamically evolve during disease progression [47–49]. This conceptual shift has redefined macrophage heterogeneity from a static classification system into a context-dependent spectrum shaped by developmental origin, microenvironmental signals, and metabolic adaptation [50,51].

Across multiple independent datasets derived from diabetic mouse models (including OVE26 and db/db mice) and human kidney biopsies, macrophages represent one of the major immune populations infiltrating diabetic kidneys [52,53]. Unsupervised clustering analyses have identified several transcriptionally distinct macrophage states, including tissue-resident macrophages (TRMs), inflammatory macrophages, interferon-responsive macrophages, proliferating macrophages, and lipid-associated macrophages (LAMs) [54]. Although these populations exhibit characteristic transcriptional signatures, increasing evidence suggests that they should not necessarily be interpreted as terminally fixed subsets. Instead, they represent dynamic cellular states occupying different positions along continuous differentiation trajectories, reflecting adaptation to the diabetic microenvironment rather than irreversible lineage commitment [50,51].

Trajectory inference approaches, including Monocle and RNA velocity analyses, further support this dynamic perspective by suggesting potential transitions among inflammatory, reparative, and metabolically adapted macrophage states during chronic disease evolution. Rather than defining discrete lineage branches, these analyses indicate gradual remodeling of transcriptional programs in response to persistent hyperglycemia, oxidative stress, lipotoxicity, and local cytokine signaling. These findings suggest that macrophage plasticity represents a fundamental feature of the diabetic renal immune microenvironment [55,56].

Recent integration of ST has added an important anatomical dimension to these observations. Distinct macrophage states exhibit preferential localization within specific pathological niches, including glomerulosclerotic lesions, inflamed tubulointerstitium, and fibrotic regions. This spatial organization suggests that macrophage phenotypes are determined not only by intrinsic transcriptional programs but also by local metabolic and inflammatory cues derived from neighboring renal parenchymal cells [49]. Consequently, macrophage heterogeneity should be interpreted as a spatiotemporally regulated process that integrates cellular identity with tissue context [57].

Collectively, evidence from single-cell atlasing has shifted the conceptual framework of macrophage biology in DKD from a binary polarization model toward a multidimensional immunometabolic landscape. This emerging paradigm provides the foundation for understanding how developmental origin, metabolic specialization, and disease progression cooperatively shape macrophage function throughout DKD.

#### Ontogeny establishes the cellular framework of macrophage heterogeneity

Developmental origin represents the first organizational layer of macrophage heterogeneity in the diabetic kidney. Independent of their transcriptional states, renal macrophages can be broadly divided into TRMs and infiltrating monocyte-derived macrophages, which differ substantially in embryonic origin, self-renewal capacity, environmental responsiveness, and contribution to disease progression [58–60].

TRMs arise predominantly from embryonic yolk sac and fetal liver progenitors and are maintained throughout adult life through local self-renewal with minimal contribution from circulating monocytes under physiological conditions [61]. These resident macrophages constitute the first line of immune surveillance within the kidney, where they continuously remove apoptotic cells, regulate tissue homeostasis, and coordinate immune tolerance through intimate interactions with TECs, endothelial cells, and stromal populations. Lineage-tracing studies have demonstrated that this embryonically established macrophage compartment remains remarkably stable during homeostasis, highlighting its fundamental role in maintaining renal immune equilibrium [59,61].

However, the diabetic microenvironment profoundly alters this homeostatic balance. Chronic hyperglycemia, oxidative stress, and sterile inflammation progressively impair TRM homeostatic functions, while simultaneously inducing robust production of chemokines such as CCL2 (MCP-1), CCL5, and CXCL10 [62,63]. These inflammatory signals promote continuous recruitment of circulating CCR2^+^ monocytes from the bone marrow, resulting in progressive expansion of infiltrating monocyte-derived macrophages within diabetic kidneys. Consequently, macrophage composition gradually shifts from a predominantly resident population toward an infiltrating inflammatory compartment as disease advances [58,63].

Importantly, developmental origin does not rigidly determine macrophage phenotype. Following renal infiltration, circulating monocytes undergo extensive environmental reprogramming driven by local metabolic stress, inflammatory cytokines, and cell–cell communication within the diabetic kidney [50,51]. As a result, macrophages sharing similar developmental origins may acquire distinct transcriptional and functional characteristics depending on their surrounding microenvironment. Conversely, resident and infiltrating macrophages may converge toward similar transcriptional states under persistent metabolic stress, further illustrating that macrophage heterogeneity reflects dynamic state transitions rather than irreversible lineage commitment [54].

Evidence supporting the pathogenic significance of monocyte recruitment extends beyond descriptive transcriptomic studies. Functional experiments using pharmacological inhibition or genetic deletion of the CCL2–CCR2 signaling axis consistently demonstrate reduced macrophage infiltration, attenuated renal inflammation, and delayed progression of tubulointerstitial fibrosis in experimental DKD [64,65]. These findings establish monocyte recruitment as a causal mechanism contributing to disease progression rather than a secondary consequence of tissue injury.

Taken together, developmental origin provides the structural framework upon which macrophage heterogeneity is established, whereas the diabetic microenvironment continuously remodels this framework through selective recruitment and environmental reprogramming. Therefore, ontogeny should be regarded as the initial layer of macrophage diversity, while subsequent immunometabolic adaptation determines the functional states ultimately observed during DKD progression.

#### Immunometabolic specialization shapes macrophage functional states

Although developmental origin establishes the cellular framework of macrophage heterogeneity, the functional diversity observed in diabetic kidneys is substantially shaped by immunometabolic specialization. Emerging evidence indicates that metabolic pathway engagement is not merely a consequence of macrophage activation but represents an important regulatory mechanism influencing macrophage fate decisions, inflammatory responses, and tissue remodeling. Rather than existing as fixed inflammatory or reparative populations, macrophages dynamically reprogram their metabolic networks in response to persistent glucotoxicity, lipotoxicity, hypoxia, and oxidative stress, thereby contributing to the emergence of distinct functional states throughout DKD progression [45,66,67].

Among these metabolic programs, enhanced glycolysis represents a prominent feature of inflammatory macrophage activation. Hyperglycemia, AGEs, damage-associated molecular patterns (DAMPs), and Toll-like receptor signaling can promote a metabolic shift from mitochondrial OXPHOS toward aerobic glycolysis [68]. This transition resembles the enhanced glycolytic metabolism observed in cancer cells and supports rapid ATP generation together with increased biosynthesis of nucleotides, amino acids, and inflammatory mediators required for sustained immune activation [69]. Concurrently, disruption of the TCA cycle results in intracellular accumulation of metabolites such as succinate and citrate. Succinate stabilizes hypoxia-inducible factor-1α (HIF-1α), thereby enhancing IL-1β production and reinforcing glycolytic metabolism through a feed-forward regulatory loop, whereas citrate provides substrates for ROS generation and inflammatory lipid mediator synthesis [70,71]. Together, these metabolic adaptations establish glycolysis as a central driver of inflammatory macrophage states rather than simply an accompanying metabolic phenotype [71].

Importantly, recent functional studies have advanced this metabolic association toward a mechanistic framework. Selective deletion or pharmacological inhibition of key glycolytic enzymes, including PKM2 and lactate dehydrogenase A, suppresses inflammatory cytokine production by macrophages and attenuates albuminuria, glomerular injury, and tubulointerstitial fibrosis in experimental diabetic models without substantially altering systemic glucose homeostasis. These findings support the concept that macrophage glycolysis is not merely a biomarker of inflammation but may represent a therapeutic vulnerability contributing to DKD progression [72].

In contrast, reparative macrophage states preferentially rely on mitochondrial OXPHOS and FAO to support sustained energy production and cellular homeostasis. These metabolic programs facilitate efferocytosis, ECM remodeling, and resolution of inflammation under physiological conditions [73,74]. However, prolonged exposure to diabetic metabolic stress progressively disrupts mitochondrial function through excessive ROS production, impaired β-oxidation, and defective mitochondrial quality control. Consequently, the capacity of reparative macrophages to maintain tissue homeostasis gradually declines, potentially increasing susceptibility to inflammatory reprogramming and impaired regenerative responses [75].

Beyond glucose metabolism, dysregulated lipid metabolism represents another important dimension of macrophage specialization in DKD. Excessive uptake of free fatty acids and modified lipoproteins can exceed mitochondrial oxidative capacity, resulting in intracellular lipid accumulation, ER stress, and mitochondrial dysfunction [50]. This imbalance establishes a maladaptive metabolic state characterized by impaired lipid utilization, defective cholesterol efflux, and persistent inflammatory activation [76]. Importantly, these lipid-associated alterations are not restricted to a single macrophage subset but represent broader adaptive responses to chronic metabolic overload that progressively reshape macrophage phenotypes during disease evolution [77].

Accumulating evidence further indicates that immunometabolic remodeling extends beyond glycolysis and lipid metabolism to include alterations in the pentose phosphate pathway, amino acid metabolism, and redox homeostasis. Activation of the pentose phosphate pathway enhances NADPH production, thereby supporting both ROS generation and antioxidant defense depending on the cellular context [78,79]. Likewise, glutamine metabolism, arginine metabolism, and mitochondrial 1-carbon metabolism collectively participate in regulating macrophage polarization, cytokine production, and epigenetic remodeling. These interconnected metabolic pathways constitute an integrated regulatory network that coordinates inflammatory signaling with cellular adaptation to metabolic stress [80,81].

Taken together, current evidence supports a conceptual shift from the traditional polarization paradigm toward an immunometabolic model of macrophage heterogeneity. Within this framework, macrophage functional states are not predefined by lineage or fixed activation programs but emerge through continuous metabolic adaptation to the evolving diabetic microenvironment. Consequently, immunometabolism serves as the central integrative axis linking environmental stress, intracellular metabolic remodeling, and macrophage functional plasticity throughout DKD progression.

#### Functional state transitions under chronic metabolic stress

While immunometabolic reprogramming establishes distinct macrophage functional states, persistent metabolic stress further drives dynamic transitions between these states during DKD progression. Rather than representing terminally differentiated populations, macrophages continuously remodel their transcriptional and metabolic programs in response to the evolving diabetic microenvironment. This remarkable plasticity enables initial adaptive responses but ultimately contributes to chronic inflammation and progressive renal fibrosis when metabolic homeostasis can no longer be maintained [45,82].

Among the metabolically adapted macrophage populations identified by single-cell transcriptomic studies, LAMs have emerged as one of the most prominent state transitions occurring during diabetic kidney injury [50]. Characterized by high expression of triggering receptor expressed on myeloid cells 2 (TREM2), apolipoprotein E, lipoprotein lipase, cathepsin B, and other lipid metabolism-related genes, LAMs expand consistently in obese and diabetic tissues across multiple independent studies [83]. Unlike inflammatory macrophages, whose primary metabolic adaptation relies on accelerated glycolysis, LAMs undergo extensive remodeling of lipid uptake, intracellular lipid trafficking, lysosomal activity, and cholesterol metabolism, representing a specialized response to chronic lipid overload [84].

Importantly, current evidence suggests that LAMs should not be regarded as an independent macrophage lineage but rather as a metabolically adaptive state induced by persistent lipotoxic stress. During the early stages of DKD, activation of the TREM2 signaling pathway enhances lipid sensing, phagocytic capacity, and cellular survival, thereby limiting excessive lipid accumulation within renal parenchymal cells and partially preserving tissue homeostasis. Functional studies further demonstrate that genetic deletion of Trem2 accelerates renal lipid deposition, exacerbates podocyte injury, and aggravates albuminuria in diabetic models, indicating that TREM2-mediated metabolic adaptation initially serves a protective role rather than simply reflecting disease severity [50,53].

However, prolonged exposure to glucolipotoxic conditions gradually exceeds the metabolic buffering capacity of LAMs. Sustained intracellular lipid accumulation induces mitochondrial dysfunction, ER stress, lysosomal impairment, and defective cholesterol efflux, collectively resulting in metabolic exhaustion [85]. Consequently, macrophages progressively lose their homeostatic functions and acquire a maladaptive phenotype characterized by increased production of inflammatory cytokines, extracellular vesicles (EVs), and profibrotic mediators. This transition illustrates how chronic metabolic stress converts an initially compensatory macrophage state into a driver of persistent tissue injury.

As DKD progresses toward advanced stages, macrophage plasticity extends beyond inflammatory regulation and directly contributes to structural remodeling of the kidney. Single-cell transcriptomic analyses have identified macrophage populations expressing profibrotic mediators, including TGF-β1, SPP1, Arg1, and ECM-associated genes, which are preferentially enriched within fibrotic renal regions [49,56,57]. Spatial transcriptomic studies further demonstrate close spatial interactions between these macrophages, activated fibroblasts, injured TECs, and endothelial cells, suggesting that macrophages actively participate in remodeling the fibrotic microenvironment rather than functioning solely as inflammatory regulators [86].

An extreme manifestation of macrophage functional plasticity is macrophage-to-myofibroblast transition (MMT), during which macrophages gradually acquire myofibroblast-like characteristics while retaining selected hematopoietic markers. Lineage-tracing experiments have confirmed that a proportion of activated myofibroblasts within fibrotic kidneys originate directly from infiltrating macrophages [87,88]. Mechanistically, activation of the TGF-β/Smad3 signaling pathway represents the principal molecular driver of MMT, whereas pharmacological or genetic inhibition of Smad3 markedly suppresses MMT formation and attenuates renal fibrosis in experimental models. More recently, EVs released from activated myofibroblasts have been shown to further enhance macrophage Smad3 activation, establishing a positive feedback circuit that perpetuates fibrogenic remodeling [89,90].

Collectively, these observations indicate that macrophage heterogeneity evolves through continuous functional state transitions rather than discrete subset replacement. Under persistent metabolic stress, macrophages progressively shift from inflammatory activation to lipid adaptation and ultimately toward profibrotic remodeling. This dynamic trajectory integrates immunometabolic adaptation with tissue remodeling and provides a mechanistic framework linking chronic metabolic dysfunction to irreversible renal fibrosis.

#### Evidence hierarchy supports a stage-dependent model of macrophage heterogeneity

Although macrophage heterogeneity has been extensively characterized by transcriptomic profiling, the strength of evidence supporting individual macrophage states varies considerably across experimental platforms. Integrating these complementary approaches establishes a hierarchical framework that progresses from descriptive cellular taxonomy to mechanistic validation and translational relevance.

At the descriptive level, single-cell RNA sequencing and single-nucleus RNA sequencing have provided the highest-resolution atlas of renal macrophage diversity, enabling identification of distinct transcriptional states and reconstruction of differentiation trajectories throughout DKD progression [49,91,92]. Integration with ST further assigns these macrophage states to defined pathological niches, thereby linking transcriptional heterogeneity to the structural organization of diabetic kidneys [57,93].

Moving beyond descriptive analyses, lineage-tracing studies provide direct evidence regarding macrophage ontogeny and cellular plasticity. These experiments demonstrate the coexistence of embryonically derived TRMs and continuously recruited monocyte-derived macrophages while simultaneously confirming macrophage contributions to myofibroblast populations through MMT [59,94]. Such findings establish developmental origin and cellular fate as fundamental determinants of macrophage diversity [88,95].

The strongest evidence supporting causal relationships derives from functional intervention studies. Conditional deletion of metabolic regulators such as PKM2, lactate dehydrogenase A, HIF-1α, Trem2, and components of the CCR2–CCL2 signaling pathway consistently alters macrophage phenotypes and significantly ameliorates renal inflammation and fibrosis in experimental DKD [72,96,97]. These investigations demonstrate that metabolic pathways actively regulate macrophage function rather than merely accompanying phenotypic changes, thereby establishing immunometabolic remodeling as a mechanistic driver of disease progression [50].

Finally, translational studies using human kidney biopsy specimens provide clinical validation of these experimental observations. Multiple independent single-cell and spatial transcriptomic datasets consistently identify inflammatory, lipid-associated, and profibrotic macrophage states within diabetic kidneys, with the abundance of these populations correlating closely with declining renal function, increasing fibrosis, and adverse clinical outcomes. Although most human studies remain observational, their remarkable consistency across independent cohorts substantially strengthens the biological relevance of macrophage immunometabolic heterogeneity in DKD [92].

Taken together, current evidence supports a unified model in which macrophage heterogeneity evolves through sequential immunometabolic state transitions during disease progression. In early DKD, inflammatory glycolytic macrophage states predominate in response to monocyte recruitment and acute metabolic stress. During intermediate stages, persistent lipotoxicity promotes the emergence of metabolically adaptive LAMs. As metabolic homeostasis progressively collapses, these adaptive states transition toward profibrotic macrophage programs that directly contribute to ECM deposition, myofibroblast activation, and irreversible renal fibrosis. This framework reconciles cellular taxonomy with functional disease progression and provides a conceptual basis for therapeutic strategies targeting macrophage immunometabolism rather than individual macrophage subsets [87] (Table 2).

**Table 2.** Immunometabolic features of macrophage states in DKD

Subtype	Marker genes	Developmental origin	Metabolic profile	Functional phenotype	Disease stage	Evidence source
Resident macrophages (TRMs)	C1qa, CX3CR1	Embryonic yolk sac / fetal liver	FAO/OXPHOS dominant	Homeostatic, immune sentinel	Early → reduced in late DKD	scRNA-seq (OVE26, human DKD)
Inflammatory macrophages	Ly6c, IL1B, TNF	Bone marrow-derived monocytes	Glycolysis ↑, TCA disruption	Proinflammatory	Early DKD	scRNA-seq^+^ functional models
IFN-high macrophages	ISG15, IFIT1	iMDMs	Glycolysis + interferon response	Antiviral/inflammatory amplification	Early/intermediate	scRNA-seq
Mrc1^+^ macrophages	Mrc1, CD206	TRM/iMDM mixed	FAO/OXPHOS	Reparative/M2-like	Intermediate	scRNA-seq
TREM2^+^ LAMs	TREM2, APOE, LPL	iMDMs + lipid-stressed TRMs	Lipid metabolism reprogramming	Lipid buffering → later dysfunction	Intermediate → late	Multi-cohort scRNA-seq
Fibrosis-associated macrophages	TGF-β1, Arg1	iMDMs	Mixed glycolysis + profibrotic program	ECM remodeling, fibroblast activation	Late DKD	scRNA-seq^+^ functional validation
Proliferating macrophages	MKI67	Local proliferation	Variable	Expansion of local pool	All stages	scRNA-seq

DKD, diabetic kidney disease; TGF-β1, transforming growth factor-β1; FAO, fatty acid oxidation; TCA, tricarboxylic acid; OXPHOS, oxidative phosphorylation; scRNA-seq, single-cell RNA sequencing; TRM, tissue-resident macrophage; TNF, tumor necrosis factor; IFN, interferon; LAM, lipid-associated macrophage; iMDM, infiltrating monocyte-derived macrophage; ECM, extracellular matrix; TREM2, triggering receptor expressed on myeloid cells 2; APOE, apolipoprotein E; LPL, lipoprotein lipase.

### Metabolic reprogramming of T cells

At the foundational observation layer of cellular infiltration and subset distribution, T cell subpopulations appear to participate in the pathophysiology of DKD by infiltrating renal tissues, modulating immune responses, and secreting cytokines [98]. The major subsets include CD4^+^ T helper (Th) cells (such as Th1, Th2, Th17, and regulatory T [Treg] cells) and CD8^+^ cytotoxic T cells. Cross-sectional analyses of peripheral blood and renal biopsy samples have generally reported increased T cell infiltration in DKD, often accompanied by an elevated CD4^+^/CD8^+^ ratio. Within these studies, proinflammatory subsets such as Th1 and Th17 are frequently enriched, whereas Treg populations tend to be reduced, suggesting a shift toward a proinflammatory immune milieu. This altered immune landscape has been associated with multiple pathological and clinical features of DKD, including proteinuria, glomerulosclerosis, interstitial fibrosis, and reduced eGFR [99,100].

At the immunopathological level, imbalance in the Th17/Treg axis has been repeatedly observed in DKD cohorts and is generally correlated with markers of renal dysfunction. An increased Th17/Treg ratio is thought to reflect an immune microenvironment characterized by enhanced IL-17-related inflammatory signaling and diminished Treg-mediated immunosuppressive activity, which may contribute to oxidative stress, podocyte injury, and renal fibrosis. However, it should be noted that the majority of existing clinical evidence is derived from relatively small-scale, cross-sectional, and often single-center studies. Sample sizes are generally limited, and external validation cohorts are rarely included. Therefore, although the Th17/Treg balance may serve as a candidate immunological indicator reflecting inflammatory status in DKD, its independent diagnostic or prognostic value remains to be confirmed in large-scale, multicenter, and longitudinal studies [98,101,102].

From a therapeutic perspective, some clinical observations suggest that interventions such as SGLT2 inhibitors may be associated with partial normalization of T cell subset distributions, including the Th17/Treg ratio, which may reflect broader restoration of immune-metabolic homeostasis. Nevertheless, whether these immunological changes represent direct causal effects or secondary consequences of improved metabolic and renal status remains unclear. Accordingly, the relationship between T cell immune remodeling and DKD progression should currently be interpreted primarily as an association rather than a proven causal axis [18,103].

Emerging experimental evidence further suggests that T cell function is closely linked to intracellular metabolic programs. For example, metabolic pathways such as glutaminolysis and glycolysis have been implicated in regulating Th17 differentiation and effector function. Preclinical studies indicate that modulation of T cell metabolic flux using small-molecule inhibitors can alter inflammatory cytokine production and may attenuate renal inflammatory responses. These findings provide mechanistic support for a model in which T cell immunometabolism contributes to immune dysregulation in DKD; however, translation of these findings into human disease remains at an early stage and requires further validation [104,105].

### Specific crosstalk between immune cells and renal-resident cells

The persistence of chronic inflammation in DKD is driven by extensive bidirectional communication between macrophages and intrinsic renal cell populations. Integrating evidence from human kidney biopsies, diabetic animal models, and emerging single-cell and spatial transcriptomic analyses, macrophage-resident cell interactions can be broadly stratified into 3 tiers of evidence strength: (a) well-established inflammatory axes, (b) experimentally supported but incompletely validated signaling pathways, and (c) emerging vesicle- and stress-associated mechanisms that require further clinical confirmation [52,106,107]. Among renal compartments, macrophage interactions with podocytes and TECs constitute the most robustly validated pathogenic networks, whereas crosstalk with mesangial, endothelial, and fibroblast populations remains comparatively less defined [108–111].

#### Macrophages and podocytes

Macrophage–podocyte communication represents one of the most extensively characterized immune-resident cell interactions in DKD. Clinical studies using human renal biopsies have demonstrated associations between macrophage accumulation, podocyte loss, and progressive glomerulosclerosis [112]. Experimental studies in diabetic models further indicate that genetic or pharmacological depletion/inhibition of macrophages can reduce podocyte injury and improve renal pathological outcomes, supporting a contributory role of macrophage-driven inflammation in DKD progression [113]. Recent spatial transcriptomic and single-cell analyses have provided additional evidence of anatomical and transcriptional proximity between macrophages and podocytes within inflammatory glomerular niches [106].

Mechanistically, macrophage–podocyte communication is largely mediated by reciprocal chemokine and cytokine signaling. Injured podocytes release CCL2 (MCP-1) and other inflammatory mediators, promoting monocyte/macrophage recruitment and activation through the CCR2 axis [106,114]. Conversely, activated macrophages aggravate podocyte dysfunction through secretion of tumor necrosis factor-α (TNF-α), IL-1β, and ROS, resulting in cytoskeletal remodeling, foot process effacement, and slit diaphragm impairment. In experimental models, macrophage-derived ROS has been shown to activate p38 mitogen-activated protein kinase signaling and enhance inflammatory cytokine production, thereby sustaining a positive feedback inflammatory loop [22,115].

Macrophage polarization provides an additional layer of regulation. Proinflammatory macrophage activation characterized by enhanced glycolytic metabolism and M1-associated cytokine production promotes podocyte injury, whereas alternatively activated macrophage states may exert protective or reparative effects under specific contexts [71,87]. Experimental studies have suggested that macrophage sirtuin 6 deficiency promotes inflammatory polarization, whereas restoration of sirtuin 6 activity or vitamin D receptor-mediated modulation favors M2-like polarization and attenuates podocyte damage; however, these findings remain largely restricted to experimental models and require validation in human DKD [116,117].

Beyond soluble inflammatory mediators, EV-mediated communication has emerged as a potential complementary mechanism. Preclinical studies suggest that macrophage-derived EVs containing specific microRNAs, including miR-21-5p, may promote podocyte inflammatory injury and pyroptotic responses, whereas EVs enriched with reparative microRNAs such as miR-25-3p or miR-93-5p may enhance autophagy-related protective pathways [4,118,119]. Nevertheless, the clinical relevance and physiological contribution of EV-mediated microRNA transfer in human DKD remain incompletely defined.

Taken together, macrophage–podocyte interaction represents a well-supported inflammatory axis in DKD, with chemokine and cytokine signaling constituting the core pathogenic framework, whereas EV-mediated regulation remains an emerging field requiring further mechanistic and clinical validation.

#### Macrophages and TECs

Macrophage–TEC crosstalk represents one of the most extensively characterized inflammatory networks contributing to tubulointerstitial injury and fibrotic remodeling in DKD. Evidence from human kidney tissues, diabetic experimental models, and single-cell/spatial transcriptomic studies demonstrates extensive bidirectional communication between these compartments, highlighting this axis as an important regulator of disease progression [52,106].

Under hyperglycemic conditions, injured TECs produce chemokines and growth factors, including CCL2 and colony-stimulating factor 1(Colony-Stimulating Factor 1), which facilitate monocyte/macrophage recruitment and activation [114,120]. Activated macrophages subsequently release proinflammatory and profibrotic mediators, including TNF-α, IL-1β, IL-6, and TGF-β1, thereby exacerbating tubular damage and promoting ECM accumulation [87,113]. This inflammatory amplification loop is further reinforced by AGEs and adhesion molecules such as intercellular adhension molecule 1, which enhance immune cell retention and inflammatory activation within the renal interstitium [99].

Metabolic and mitochondrial stress further intensify macrophage–TEC interactions. In experimental models, mitochondrial injury in macrophages promotes the release of mitochondrial DNA, which activates TLR9–MyD88-dependent inflammatory signaling and enhances oxidative stress responses [121]. Conversely, stressed or ferroptotic TECs may release DAMPs, including HMGB1 and extracellular ATP, which activate macrophage inflammatory pathways involving TLR4 and purinergic P2X7 signaling [46,122]. These reciprocal stress responses may establish a self-reinforcing inflammatory circuit that sustains tubular injury, although their direct contribution to human DKD requires further validation.

EV-mediated communication represents an emerging layer of macrophage–TEC regulation. Experimental studies have shown that TEC-derived EVs enriched in LRG1 can promote macrophage activation through TGF-β receptor signaling, whereas macrophage-derived EVs carrying TRAIL may induce tubular epithelial apoptosis [123]. In addition, Dll4-containing exosomes have been reported to regulate macrophage activation and NF-κB signaling through Notch-related pathways [124]. However, these EV-based mechanisms remain largely supported by experimental evidence, and their clinical relevance in human DKD remains incompletely defined.

Collectively, macrophage–TEC interactions represent a highly characterized inflammatory axis in DKD, integrating chemokine-mediated recruitment, metabolic stress responses, and emerging EV-based intercellular communication.

#### Macrophages and mesangial cells

Compared with podocyte and TEC interactions, macrophage–mesangial communication is less extensively characterized and remains supported predominantly by experimental studies. Nevertheless, accumulating evidence suggests that this interaction may contribute to glomerular inflammation, mesangial activation, and ECM expansion during DKD progression [87,99,113].

Under diabetic conditions, mesangial cells exposed to hyperglycemic and inflammatory stimuli produce chemokines and cytokines that may facilitate macrophage recruitment and activation [4]. Activated macrophages subsequently release proinflammatory and profibrotic mediators, including TNF-α, IL-1β, and TGF-β1, which promote mesangial cell activation, proliferation, and ECM accumulation, thereby contributing to glomerulosclerotic remodeling [84,125]. This reciprocal signaling may establish a feed-forward inflammatory circuit; however, its contribution to human DKD progression remains incompletely established.

Emerging evidence suggests that EV-mediated communication may represent an additional regulatory mechanism. Experimental studies indicate that macrophage-derived EVs can influence mesangial cell activation through profibrotic pathways involving TGF-β1/Smad signaling, thereby promoting matrix deposition [126]. However, these findings are primarily derived from in vitro systems and rodent models, limiting direct translation to human DKD.

Compared with macrophage interactions involving podocytes or TECs, macrophage–mesangial communication remains relatively underdefined, and its precise contribution to DKD progression requires further validation using human biopsy studies and spatially resolved approaches.

#### Macrophage interactions with endothelial cells and fibroblasts

Macrophage interactions with endothelial cells and fibroblasts are increasingly recognized as potential contributors to microvascular rarefaction and fibrotic remodeling in DKD, although supporting evidence remains largely derived from preclinical studies.

Endothelial cells exposed to hyperglycemic stress undergo functional and transcriptional alterations involving HIF-1α–Notch-related signaling pathways, which may facilitate macrophage recruitment and inflammatory activation. These reciprocal interactions contribute to endothelial dysfunction, capillary rarefaction, and persistent hypoxic stress, thereby promoting renal injury progression. Pharmacological activation of peroxisome proliferator-activated receptor-α has been shown to ameliorate endothelial dysfunction and inflammatory responses in experimental diabetic models, suggesting potential vascular protective effects [127].

Within the fibrotic niche, macrophage-derived TGF-β1 promotes fibroblast activation and ECM deposition, representing a major mechanism linking chronic inflammation to renal fibrosis. In addition, macrophage-associated galectin-3 enhances TGF-β receptor signaling and increases fibroblast responsiveness, thereby accelerating interstitial fibrotic remodeling [88,128]. These findings suggest that macrophages may serve as important regulators of the transition from chronic inflammation to fibrosis in DKD.

**Fig. 5.**
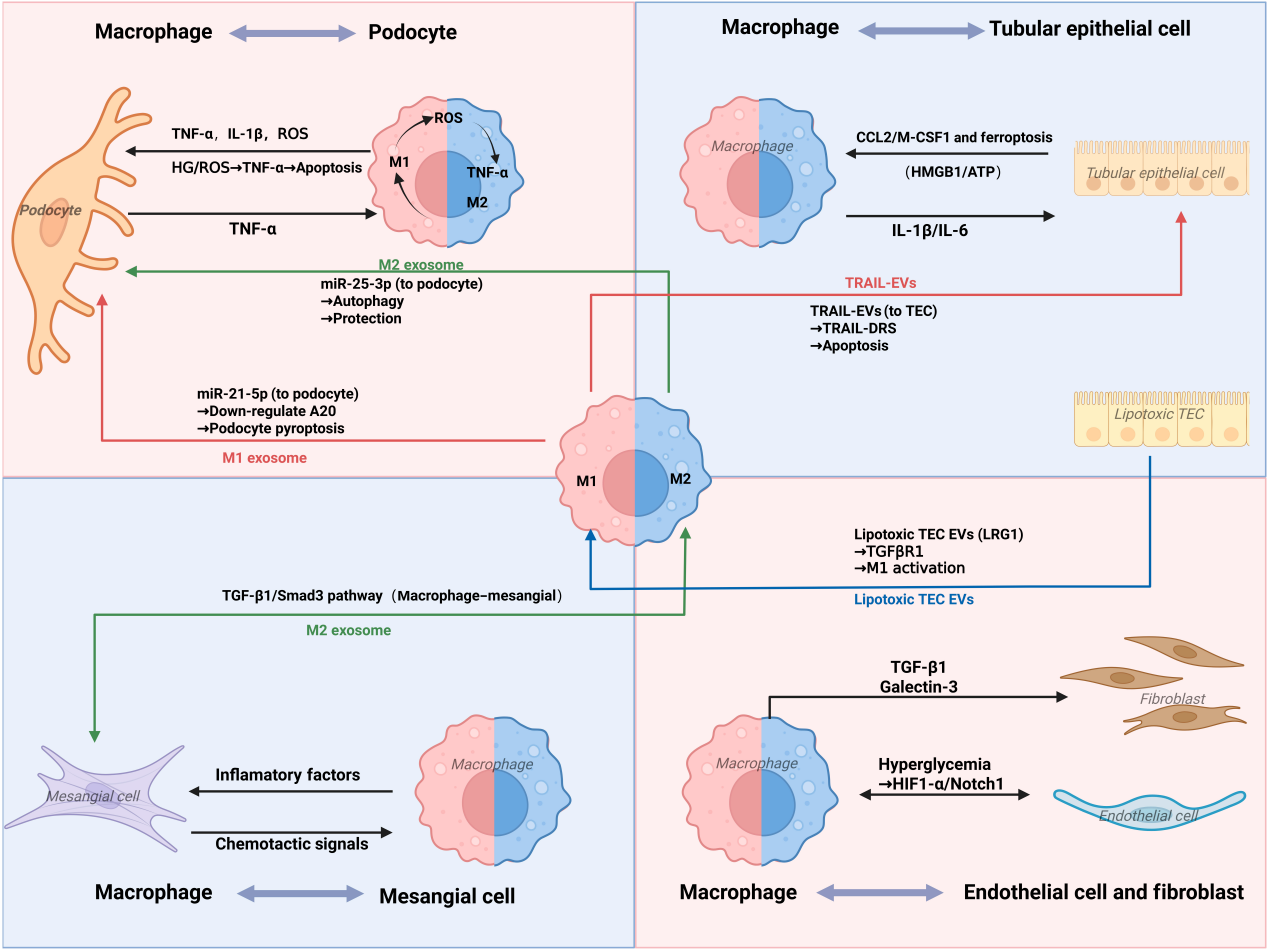
Specific interactions between immune cells and resident renal cells. The specific interaction between the intrinsic cells of the kidney and immune cells is the key to the persistent inflammation in diabetic kidney disease (DKD). Macrophages have a close bidirectional interaction with the intrinsic cells of the kidney (podocytes, renal tubular epithelial cells, mesangial cells, and endothelial cells). Figure created with BioRender.com.

Despite these insights, most available evidence originates from animal models or in vitro studies, and direct validation in human DKD tissues remains limited. Therefore, macrophage–endothelial cell and macrophage–fibroblast interactions should currently be considered emerging contributors to DKD pathophysiology, with increasing mechanistic understanding but incomplete translational confirmation (Fig. 5).

### Interorgan crosstalk

Within the macroscopic theoretical framework of organ communication, the gut–kidney axis plays a pivotal role in the immunomodulation of DKD, constituting a bidirectional pathophysiological feedback loop. The current conceptual framework proposes that, on one hand, declining renal function leads to systemic accumulation of uremic toxins, which may directly alter gut microbial composition and impair beneficial microbial functions. On the other hand, gut dysbiosis may compromise intestinal epithelial barrier integrity, increasing the translocation of endotoxins into the systemic circulation. This process subsequently triggers persistent systemic inflammation, thereby exacerbating renal injury and fibrogenesis [129].

At the empirical level of microbiomics and metabolomics, patients with DKD exhibit profound gut dysbiosis, characterized by reduced abundance of short-chain fatty acid (SCFA)-producing bacteria (e.g., *Bifidobacterium*) and enrichment of uremic toxin-producing bacteria (e.g., *Clostridium*) [130,131]. Descriptive evidence indicates that SCFA levels positively correlate with eGFR, whereas reduced SCFA availability is associated with elevated HbA1c levels and insulin resistance, potentially contributing to impaired glycemic control. Conversely, alterations in uremic toxin profiles are associated with inflammatory activation and fibrotic progression. Mechanistic studies have further revealed the biological basis underlying intestinal barrier disruption. This dysbiosis is exacerbated under diabetic conditions; specifically, impaired intestinal barrier function (e.g., loss of MAVS protein) facilitates the translocation of opportunistic pathogens (e.g., *Klebsiella pneumoniae*) into the bloodstream and promotes local intestinal IL-17 production. These circulating bacterial components, together with IL-17-mediated immune activation, may amplify systemic inflammation and intensify renal inflammatory responses and fibrosis [132].

To move beyond descriptive microbial alterations and establish potential causality, recent fecal microbiota transplantation studies have provided important experimental evidence. Transplantation of dysbiotic microbiota from DKD patients into antibiotic-depleted, diabetes-susceptible mice not only induced intestinal barrier disruption in recipient animals but also aggravated glomerular mesangial expansion and proteinuria. These findings support a contributory role of gut microbial alterations in renal injury rather than representing merely a passive consequence of the uremic environment [133,134]. Beyond microbial composition itself, microbiota-derived metabolites serve as critical mediators of gut–kidney communication. At the translational interface linking molecular mediators with clinical phenotypes, beneficial microbial metabolites, such as SCFAs, contribute to the maintenance of renal homeostasis, whereas dysbiosis-associated signatures, including enrichment of specific gram-negative bacterial genera and increased production of trimethylamine N-oxide, are associated with inflammatory activation and metabolic disturbances in CKD and DKD [135,136]. These microbiota-related alterations correlate with clinical parameters, including eGFR and systemic inflammatory markers. However, although these biomarkers demonstrate strong associations in cross-sectional studies, prospective longitudinal investigations are still required to determine their independent predictive value for DKD progression and staging [137,138].

Importantly, recent longitudinal interventional studies have shown that supplementation with specific butyrate-producing bacteria or exogenous SCFAs can attenuate activation of intrarenal NLRP3 inflammasome signaling. This intervention-based evidence further supports the functional importance of the gut–kidney axis in immunometabolic regulation. Consequently, longitudinal monitoring of fecal and circulating microbial-derived markers, combined with targeted interventions such as probiotics or metabolite-based therapies, may provide translational opportunities for improving clinical outcomes, including eGFR and urinary albumin-to-creatinine ratio [135,136,139].

### The renal immuno-stromal interaction network: From spatial association to mechanistic validation in DKD immunometabolism

A central unresolved question in DKD immunometabolism is how local metabolic disturbances within renal parenchymal cells are translated into spatially organized immune activation and ultimately contribute to progressive structural remodeling. In particular, it remains unclear how lipid overload, hypoxia, and glucotoxicity stress within tubular and glomerular compartments are coupled to immune cell recruitment, activation, and persistence within defined microanatomical niches. Emerging spatial and organoid technologies are increasingly being applied not only to map these processes but also to interrogate the potential causal relationships between metabolic reprogramming and immune remodeling in situ [77,93,140].

From the perspective of lipid-driven inflammation, desorption electrospray ionization mass spectrometry imaging (DESI-MSI) enables high-resolution spatial mapping of lipid species within kidney tissues. Beyond simple colocalization, DESI-MSI-based studies have demonstrated that regions of altered lipid accumulation are spatially associated with inflammatory microenvironments enriched in immune cells, particularly in areas of tubular injury [141]. Mechanistically, these spatial relationships support the hypothesis that tubular lipid overload may serve as a metabolic stressor promoting local macrophage recruitment and activation, thereby contributing to the establishment of lipotoxic inflammatory niches. However, because these observations remain largely correlative, they generate mechanistic hypotheses rather than establish direct causality.

To address this limitation, ST combined with ligand–receptor inference approaches, such as Cell Chat, has enabled more detailed investigation of intercellular signaling within defined renal microdomains. These analyses have identified activation of chemokine-associated communication pathways, including CCL2–CCR2 signaling, between injured TECs and infiltrating macrophages in renal disease models [142,143]. Importantly, these findings provide a mechanistic link between tubular stress responses and immune cell recruitment, suggesting that metabolic alterations in tubular cells may actively shape the immune microenvironment through paracrine signaling. In this context, TECs may function as metabolic sensors that translate intracellular stress into immune-modulating signals, thereby amplifying local inflammation and progressive renal injury.

To move beyond spatial inference toward functional validation, patient-derived kidney organoids generated from DKD-related induced pluripotent stem cells have emerged as experimentally tractable platforms for investigating renal metabolic and immune interactions. Compared with conventional 2-dimensional cultures, organoids preserve aspects of renal cellular organization and allow perturbation-based evaluation of mechanisms underlying metabolic stress responses. These systems may facilitate investigation of whether hyperglycemia, lipid overload, or circulating metabolic factors directly regulate chemokine production and immune cell recruitment within nephron-like structures. Furthermore, organoid platforms provide opportunities to explore how systemic metabolic mediators, including gut-derived metabolites such as trimethylamine N-oxide and SCFAs, influence renal immunometabolism communication and macrophage functional states [144].

At the whole-organ level, advances in light-sheet fluorescence microscopy combined with tissue-clearing approaches, including Clarity-based methods, have enabled 3-dimensional visualization of immune, vascular, and stromal networks within intact organs. These approaches reveal that immune cell localization is highly organized and influenced by anatomical structures rather than randomly distributed. Emerging studies suggest that alterations in neuroimmune and stromal interactions may contribute to chronic inflammatory activation in metabolic kidney diseases, including DKD [145,146]. Rather than representing merely descriptive neuroanatomical changes, these observations raise the possibility that metabolic stress disrupts neuropeptide-mediated immune regulatory circuits, thereby increasing macrophage activation thresholds and sustaining inflammation. Functional denervation and neuromodulator intervention studies provide preliminary support for this concept; however, direct causal validation in DKD remains limited [147–149].

Collectively, these complementary technologies establish a hierarchical framework for investigating DKD immunometabolism: DESI-MSI defines spatial metabolic abnormalities, ST-Cell Chat identifies candidate intercellular communication pathways, organoid systems enable perturbation-based mechanistic testing, and light-sheet fluorescence microscopy-based imaging contextualizes these processes within intact tissue architecture. Together, these approaches shift the field from static spatial association toward experimentally testable models of metabolic-immune interaction and provide a foundation for identifying therapeutically actionable mechanisms in DKD [93].

## Targeted Intervention Strategies Based on Pathological Mechanisms

The pathological mechanisms underlying DKD are highly intricate, involving multidimensional interactions among hemodynamic abnormalities, metabolic reprogramming, oxidative stress, and dysregulation of the immune microenvironment [150,151]. Conventional therapeutic strategies have primarily focused on glycemic and blood pressure control. However, with the deepening understanding of DKD at the molecular level, targeted interventions aimed at specific pathogenic processes—particularly mitochondrial dysfunction, immunometabolism reprogramming, and aberrant intercellular communication—have emerged as critical avenues for improving patient outcomes. As current intervention strategies undergo a paradigm shift from “broad-spectrum control” to “mechanism-driven” approaches, this chapter categorizes interventions into clinically validated standardized therapies, drug candidates currently in clinical trials, and preclinical frontier strategies at the stage of proof of concept, in order to clearly define the clinical translational degree of each technology [32].

### Clinically validated standardized therapies: Amelioration of hemodynamic and metabolic disturbances

For patients with early-stage DKD, particularly those exhibiting persistent hyperglycemia, RAAS activation, and glomerular hemodynamic abnormalities, therapeutic strategies are currently dominated by guideline-endorsed interventions supported by large-scale randomized controlled trials. These therapies have undergone large-scale phase III clinical evaluation and represent the current standard of care rather than experimental approaches.

SGLT2 inhibitors represent the most extensively validated renoprotective therapies in DKD. Large-scale trials, including DAPA-CKD and EMPA-KIDNEY, demonstrated significant reductions in kidney disease progression, including sustained eGFR decline and kidney failure events. These benefits are largely mediated through restoration of tubuloglomerular feedback, reduction of intraglomerular pressure, and modulation of inflammatory and fibrotic pathways. Importantly, the renal benefits of SGLT2 inhibitors extend beyond glucose lowering, supporting their central role in contemporary DKD management [152–154].

Glucagon-like peptide-1 receptor agonists (GLP-1RAs) have emerged as guideline-supported adjunctive therapies with increasing evidence for renal protection. Data from cardiovascular outcome trials and the FLOW trial indicate that GLP-1RA therapy reduces albuminuria progression and improves kidney outcomes, although their renal protective effects remain less extensively validated compared with SGLT2 inhibitors [155,156].

Nonsteroidal mineralocorticoid receptor antagonists, particularly finerenone, provide another evidence-based therapeutic option for patients with albuminuric DKD receiving optimized RAAS blockade. The FIDELIO-DKD and FIGARO-DKD trials demonstrated significant reductions in kidney and cardiovascular composite outcomes, with benefits potentially related to inhibition of mineralocorticoid receptor-mediated inflammatory and fibrotic responses [157,158].

Collectively, SGLT2 inhibitors, GLP-1RAs, and nonsteroidal mineralocorticoid receptor antagonists represent clinically mature interventions supported by phase III evidence and guideline recommendations. In contrast, immunometabolic modulators, targeted nano therapies, and cell-based approaches remain investigational and require further translational validation.

### Mechanism-targeted therapeutics under clinical development: Oxidative stress and metabolic homeostasis

Beyond established therapies, several mechanism-driven agents are currently undergoing clinical evaluation. These strategies aim to directly target pathogenic pathways implicated in DKD progression, including lipid accumulation, oxidative stress, mitochondrial dysfunction, and inflammatory activation. However, their clinical efficacy remains to be fully established.

1. Lipotoxicity and vascular injury repair: To counteract the overexpression of VEGF-B induced by chronic hyperglycemia, investigators developed the monoclonal antibody CSL346. This agent is designed to block VEGF-B-mediated ectopic lipid deposition within the kidney and subsequent development of insulin resistance. Currently being evaluated in clinical trials, this strategy aims to restore podocyte function and mitigate lipid-induced renal injury [159].
2. Amelioration of metabolic syndrome and podocyte homeostasis: INV-202, a peripheral cannabinoid receptor 1 inverse agonist, modulates metabolic reprogramming, attenuates renal fibrosis, and preserves glomerular podocyte structural proteins, including podocin and nephrin. Early clinical studies suggest potential metabolic and renoprotective benefits; however, definitive evidence from renal outcome trials remains unavailable. Further investigation is required to determine whether this agent can effectively modulate metabolic reprogramming and attenuate fibrotic progression [160].
3. Alleviation of ER stress: Farnesoid X receptor agonists, exemplified by nidufexor, modulate bile acid metabolism and enhance expression of molecular chaperones such as binding immunoglobulin protein, thereby potentially alleviating renal injury induced by metabolic stress. Having completed selected phase II clinical studies, this compound requires further evaluation to determine its clinical efficacy in regulating bile acid homeostasis and reducing metabolic stress [161].
4. Upstream inhibition of ROS generation: Isuzinaxib (APX-115), a novel NOX inhibitor, and TMX-049, a xanthine oxidoreductase inhibitor, selectively target NOX and xanthine oxidase, respectively, to reduce superoxide generation at an early stage. Both investigational agents are currently under clinical development and have demonstrated preliminary signals of potential proteinuria reduction [162,163].
5. Blockade of upstream inflammatory cascades: Targeting DAMPs, the anti-IL-33 monoclonal antibody tozorakimab has been developed to disrupt the IL-33/ST2 signaling axis. By interfering with this upstream inflammatory amplification pathway, this therapeutic approach aims to suppress immune cell activation and recruitment and is currently undergoing clinical evaluation [164].

### Preclinical proof-of-concept strategies: Immunometabolic remodeling and frontier technologies

In contrast to clinically validated pharmacological approaches, these strategies remain largely restricted to experimental models. Although they provide important mechanistic insights into immunometabolic regulation, their translation is limited by challenges including delivery specificity, safety evaluation, manufacturing scalability, and long-term efficacy.

1. Macrophage immunometabolic reprogramming: Functionalized nanocarriers conjugated with F4/80 antibodies have been developed to specifically deliver the glycolytic inhibitor 2-deoxy-D-glucose into intrarenal M1-like macrophages. This targeted delivery strategy suppresses excessive glycolytic activity and reduces the metabolism-driven release of proinflammatory mediators [165].
2. Adaptive ROS scavenging and ferroptosis inhibition: A nanotechnology-based “selenium-embedded adaptive antioxidant nanomedicine” has been engineered to directly scavenge ROS and release elemental selenium in response to oxidative stimuli. The released selenium subsequently promotes de novo synthesis of glutathione peroxidase 4, a key ferroptosis-regulating enzyme, thereby improving mitochondrial antioxidant capacity and reducing lipid peroxidation-driven ferroptosis [166].
3. Modulation of T cell subset homeostasis: Mesenchymal stem cell-based therapies (such as ORBCEL-M) exert immunomodulatory effects through paracrine signaling pathways and direct cell-to-cell interactions. These therapeutic approaches promote expansion of Treg cells while suppressing pathogenic natural killer T cells and inflammatory monocytes, thereby contributing to restoration of immune homeostasis. Although early clinical investigations have been initiated, these approaches remain in an exploratory translational stage due to substantial challenges, including product standardization, manufacturing complexity, and cost control [167] (Table 3).

**Table 3.** The therapeutic mechanism and target sites of DKD

Transformation level	Target dimension	Representative drug/technology	Core mechanism of action	Clinical stage	Evidance source	Renal end points	Major translational barriers
Standard treatment	Tubuloglomerular feedback, intraglomerular pressure, inflammation	SGLT2 inhibitors	Restore tubuloglomerular feedback; reduce intraglomerular pressure and inflammation	Approved; guideline-recommended	DAPA-CKD, EMPA-KIDNEY (Phase III RCTs)	eGFR decline, kidney failure, CKD progression, albuminuria	Residual renal risk; variable response
	Metabolic regulation, inflammation, oxidative stress	GLP-1RAs	Improve metabolic homeostasis; reduce inflammation and oxidative stress	Approved; expanding renal indications	CV outcome trials; FLOW trial	Albuminuria, eGFR slope, kidney composite outcomes	Renal outcome evidence less mature
	Mineralocorticoid receptor signaling, inflammation, fibrosis	Finerenone (nsMRA)	Block mineralocorticoid signaling; attenuate inflammation and fibrosis	Approved; add-on therapy	FIDELIO-DKD, FIGARO-DKD (Phase III RCTs)	Kidney failure, eGFR decline, albuminuria	Hyperkalemia; residual fibrosis
In clinical development	Lipotoxicity and vascular damage	CSL346 (VEGF-B monoclonal antibody)	Reduce ectopic lipid accumulation and metabolic injury	Phase I/II	Clinical studies; preclinical models	Albuminuria, renal function markers	Lack of phase III renal outcomes
	Podocyte homeostasis and metabolic reprogramming	INV-202 (CB1R inverse agonist)	Modulate metabolism and reduce fibrosis/podocyte injury	Phase I/II	Clinical studies; experimental models	Albuminuria, eGFR, fibrosis markers	Long-term efficacy and safety
	ER stress	Nidofosol (FXR agonist)	Regulate bile acid metabolism and ER stress response	Phase II	Clinical studies; preclinical models	Proteinuria, renal biomarkers	Limited kidney-specific validation
	Oxidative stress	Isuzinaxib (a NOX inhibitor) and TMX-049 (an XOR inhibitor)	Inhibit NOX/XOR-mediated ROS production	Phase I/II	Clinical studies; preclinical models	Proteinuria, oxidative stress markers	Durable renal benefit remains unclear
	Upstream inflammatory signals	Tocilizumab (an inhibitor of IL-33)	Block IL-33/ST2 inflammatory signaling	Clinical development	Clinical trials; experimental DKD evidence	Renal inflammation, albuminuria (under evaluation)	DKD-specific efficacy unknown
Preclinical exploration	Macrophage metabolic reprogramming	F4/80 conjugated nanocarriers loaded with 2-DG	Suppress macrophage glycolytic reprogramming and inflammation	Preclinical	DKD animal models	Albuminuria, inflammation, fibrosis	Delivery safety and scalability
	Antioxidation and inhibition of ferroptosis	Selenium-embedded adaptive nano-drug (AAN)	Enhance GPX4 activity; inhibit ROS-driven ferroptosis	Preclinical	DKD animal models	Oxidative injury, mitochondrial dysfunction, fibrosis	Pharmacokinetics and translation
	Immune balance regulation	Mesenchymal stem cell therapy (such as ORBCEL-M)	Restore immune balance via Treg induction and immune modulation	Early translational stage	Cell therapy studies; experimental models	Renal function, inflammation, fibrosis	Standardization and cost

DKD, diabetic kidney disease; Treg, regulatory T; SGLT2, sodium–glucose cotransporter 2; CKD, chronic kidney disease; eGFR, estimated glomerular filtration rate; ER, endoplasmic reticulum; RCT, randomized controlled trial; nsMRA, nonsteroidal mineralocorticoid receptor antagonist; CBIR, cannabinoid receptor 1; FXR, farnesoid X receptor; 2-DG, 2-deoxy-D-glucose; AAN, adaptive antioxidant nanomedicine; GPX4, glutathione peroxidase 4; NOX, NADPH oxidase; IL-33, interleukin-33

## Conclusion and Future Perspectives

While substantial advances in the understanding of DKD pathogenesis and therapeutic development have facilitated the emergence of novel pharmacological agents and treatment paradigms, successful clinical translation remains challenging. Current therapies are still limited by predominantly single-target mechanisms, substantial interindividual variability in treatment responses, and incomplete understanding of long-term safety profiles. Meanwhile, emerging approaches based on stem cell therapy and nanotechnology remain largely confined to preclinical studies or early-stage clinical evaluation. Key challenges, including long-term efficacy, scalable biomanufacturing, and precise spatiotemporal control of therapeutic delivery, require rigorous validation. Furthermore, the frequent failure of promising preclinical candidates to achieve primary renal end points in clinical trials highlights the complex and multifactorial nature of DKD pathogenesis, which remains incompletely understood.

Future efforts should prioritize the integration of phenotypic stratification with precision therapeutic strategies. Multi-omics approaches combined with advanced bioinformatics will be essential for resolving molecular heterogeneity in DKD and facilitating the development of individualized treatment strategies. From a therapeutic perspective, promising directions include the identification of novel targets involved in oxidative stress, metabolic reprogramming, and immune dysregulation; the development of kidney-targeted delivery platforms; and the establishment of rational combination therapies addressing multiple pathogenic pathways. Ultimately, overcoming current therapeutic limitations in DKD will require interdisciplinary collaboration and continuous bidirectional communication between fundamental discoveries and clinical translation.
